# Use of peripherally inserted central venous catheters and midline catheters for palliative care in patients with cancer: a systematic review

**DOI:** 10.1007/s00520-024-08664-3

**Published:** 2024-06-26

**Authors:** Eva Gravdahl, Dagny Faksvåg Haugen, Olav Magnus Fredheim

**Affiliations:** 1 Department of Palliative Medicine, Akershus University Hospital, Sykehusveien 25, 1478, Lørenskog, Norway; 2 Regional Centre of Excellence for Palliative Care, Western Norway, Haukeland University Hospital, Bergen, Norway; 3 Department of Clinical Medicine K1, University of Bergen, Bergen, Norway; 4 Institute of Clinical Medicine, University of Oslo, Oslo, Norway

**Keywords:** Cancer, Drug administration, Palliative care, Intravenous access, Peripherally inserted central catheter, Midline catheter

## Abstract

**Background:**

Peripherally inserted central catheters (PICCs) and midline catheters (MCs) may offer convenient intravenous access, but evidence to support their place in palliative care is limited. This review aimed to assess catheter indications, utilization, complications, dwell time, and patient experiences in cancer patients receiving palliative care.

**Methods:**

A systematic search for studies on catheter utilization for supportive or symptom treatment was conducted in Medline, Embase, CINAHL, Web of Science, Cochrane, and CENTRAL databases. Studies with a study population or a subgroup of palliative care cancer patients were included. Study quality was assessed using the Effective Public Health Practice Quality assessment tool.

**Results:**

Of 7631 unique titles, 17 articles were examined in detail, all published between 2002 and 2022. Median catheter dwell time varied from 15 to 194 days, the longest when utilized for home parenteral nutrition. For pain and symptom management, the typical duration was 2–4 weeks, often until the patient’s death. Complication rates were minimal, with thrombosis, infections, and occlusion ranging from 0 to 2.46 incidents per 1000 catheter days. In studies from palliative care services, patients reported minimal distress during procedures and high user satisfaction. Quality of life assessments post-procedure improved, possibly influenced by concurrent specialist palliative care provision. All studies were assessed to be of moderate or weak quality.

**Conclusion:**

PICC and MC are safe and valuable tools in palliative care cancer patients who would benefit from intravenous access for symptom management. Further studies are needed to clarify indications for PICC or MC in palliative care.

**Supplementary Information:**

The online version contains supplementary material available at 10.1007/s00520-024-08664-3.

## Introduction

Traditionally, the subcutaneous (SC) route has due to its simplicity, safety, and ubiquity been preferred for parenteral drug administration in palliative care patients. This is reflected in existing guidelines where the SC route is advocated as the preferred route for parenteral administration [1, 2]. Yet, the intravenous (IV) route, with its broader applicability, reduced incidence of local infiltrations and irritations, and more consistent pharmacokinetics, is recognized as a valuable alternative, especially when rapid drug action is paramount [3, 4].

Peripherally inserted central venous catheters (PICC) and midline catheters (MC) not only enable easier IV treatment in home care settings but also sidestep the discomforts associated with frequent venipuncture and cannulation [5, 6]. PICCs are inserted into the basilic, cephalic, or brachial vein by percutaneous cannulation. MCs, peripheral intravenous lines ranging between 3 and 8 inches, are inserted by the same technique without advancing the catheter beyond the distal axillary vein, primarily catering to shorter-term requirements.

While several studies address PICC placement in cancer patients undergoing chemotherapy, comprehensive guidelines from both European and American Societies of Oncology neither endorse nor discourage the use of a particular type of central venous catheter [7, 8]. Notably, recent randomized controlled trials support the preference of venous access ports over PICC for chemotherapy due to thrombosis concerns [9, 10]. The Michigan Appropriateness Guide for Intravenous Catheters (MAGIC), based on a combination of systematic reviews and expert opinions, positions PICCs as a viable option for cancer patients undergoing prolonged IV therapy [11]. However, it underscores a paucity of data concerning palliative care.

The aim of this systematic review was to comprehensively assess the use of PICC and MC in palliative care cancer patients. This entails identifying the indications and settings for catheter placement, treatments and procedures in which these catheters are applied, catheter dwell times, the timing of placement in relation to a patient’s death, and complications and reasons for catheter removal. Additionally, this review aimed to assess palliative care cancer patients’ experiences with PICC or MC.

## Method

The study was conducted following PRISMA guidelines [12]. A systematic search was conducted in Medline, Embase, CINAHL, Web of Science, Cochrane, and CENTRAL databases. The search strategy to identify palliative care patients was adapted from a validated search filter for identifying this patient population [13]. The complete search strings (Appendix 1) were developed by an experienced biomedical librarian specialist with input from the researchers. The last day searched was December 23rd, 2022. Additionally, reference lists of the included articles were hand-searched for relevant papers.

Titles and abstracts of the retrieved citations were reviewed by one of the researchers (EG), and potentially relevant papers were read in full text by two researchers (EG, OMF). In cases of doubt or disagreement, papers were reassessed by EG and OMF to reach an agreement.

### Inclusion criteria

Papers were selected for further reading if the abstract contained any information related to the use of PICC or MC for supportive or symptom treatment in patients with cancer receiving palliative care. Full-text publications that identified a group or subgroup of cancer patients and described catheter utilization for palliative care interventions were included in the review. Palliative care interventions included any pain or other symptom treatment, transfusions, hydration, and parenteral nutrition. Studies on patients with a catheter primarily for administration of tumor-targeted treatment were not included. Studies with a heterogeneous study population were included if the majority of patients were cancer patients receiving palliative care or if data from a subgroup matching this description were presented separately. In line with the WHO definition of palliative care [14], studies on cancer patients receiving palliative care were included irrespective of expected survival time. Accordingly, study populations range from end-of-life care to patients with a longer life expectancy.

The review incorporated studies that employed either an experimental or quasi-experimental design (including clinical trials, cohorts, or case–control studies), as well as case reports, cross-sectional studies, and literature reviews. Studies addressing patient experiences were included. Any comparison groups, or the lack thereof, were accepted. Opinion pieces and conference abstracts were excluded. Studies were restricted to English language and publication date of January 2000 or later.

### Quality assessment

The Effective Public Health Practice Quality (EPHPP) assessment tool for quantitative studies [15] was used to rate the 17 studies. This tool is applicable across a wide range of quantitative study designs. Studies were rated weak, moderate, or strong on the following six components: selection bias, study design, confounders, blinding, data collection, and withdrawal. The quality ratings across the six domains were aggregated to give a global rating for each study as follows: weak (two or more component weak ratings), moderate (one weak rating), or strong (no weak ratings). Quality assessments were scored independently by two authors (EG, OMF), and discrepancies were discussed until a consensus was reached. Bias was further discussed at an outcome level when considered relevant.

## Results

### Abstracts and papers

**Fig. 1:**
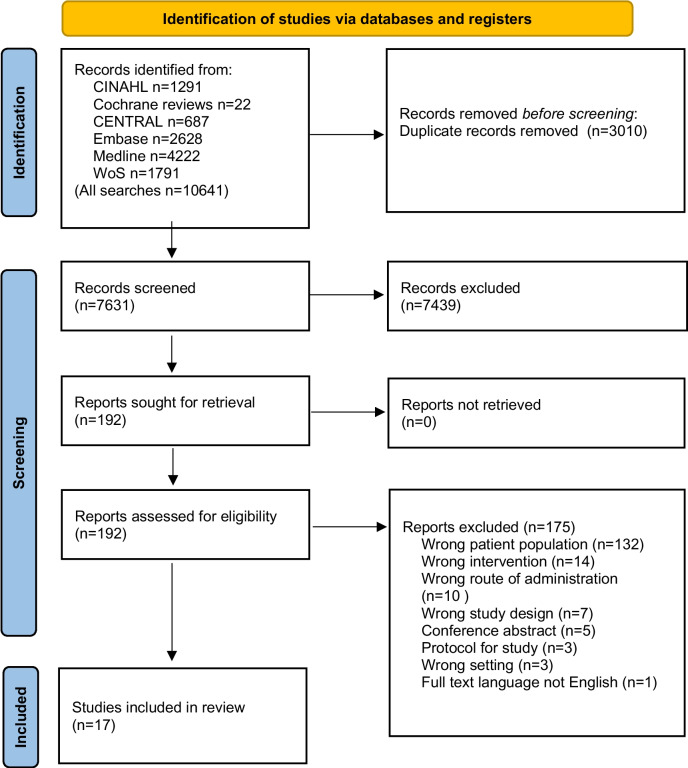
PRISMA flow diagram of study selection

The search identified 4222 titles in Medline, 2628 in Embase, 1791 in Web of Science, 1291 in CINAHL, 687 in CENTRAL, and 22 in the Cochrane database (Fig. 1). After the removal of duplicates, a total of 7631 titles and abstracts were screened, yielding 192 articles that were selected for further reading according to the selection criteria described above. After examining the full papers, 17 articles were included in the study. Hand searching the reference lists of included papers did not identify additional relevant articles. Heterogeneity of studies did not allow for meta-analysis.

The 17 studies included in the analysis were published between 2002 and 2022 (Table 1). Eight were conducted in Europe (Italy, Poland, Spain, Switzerland, UK) [5, 6, 16–21], three in the USA [11, 22, 23], and six in Asia (China, Japan, South Korea, Taiwan) [24–29]. All studies were conducted either exclusively or predominantly in patients with a main diagnosis of cancer. One study was a randomized controlled trial [26], one a systematic review with guideline [11], four cohort studies [16, 18, 19, 22], nine case series [5, 6, 17, 20, 21, 23, 24, 27, 28], and two single case reports [25, 29]. Nine of the in total 13 case series and cohort studies were prospective surveys.

**Table 1:** PICC and MC for palliative care cancer patients

First author, year, country	Title	Study design, *N* total	Population	PICC/MC/comparison	PICC or MC catheter dwell time (days)	Catheter removal reasons	PICC/MC complications (%)	Survival after placement	Catheter-related complications/1000 catheter days	Main conclusion	EPHPP global rating
Walshe 2002, USA [23]	Complication Rates Among Cancer Patients With Peripherally Inserted Central Catheters	Case series (prospective) *n* = 351 catheters 335 unique patients	All cancer patients (adults and children) at a tertiary care cancer center who had a PICC successfully inserted during a 1-year period	PICC	Median (range) 15 (1–487)	Completed therapy 39%, death 21.9%, complications 32.8%, CRBSI 7.4%, phlebitis 6.6%, DVT 3.4%, leakage 6%, accidental removal 5.4%	32.8	NR 22% of PICC removals within a 2-year follow-up period were due to patient death	CRBSI 2.46, phlebitis 2.18, thrombosis 1.14, occlusion 1.33, leakage 1.99, accidental dislocation 1.8	PICCs placed into patients with cancer have a high overall complication and infection rate approximating patients with tunneled central venous devices. The convenience and simple management of complications argue for continued widespread PICC use in the cancer population	Weak
Lin 2010, Taiwan [24]	The Use of Peripherally Inserted Central Catheters in Cancer Patients	Case series (retrospective chart review) *n* = 568 catheters 524 unique patients	Cancer patients in need of PICC for chemotherapy or terminally ill cancer patients in need of hospice care	PICC for terminal care (*n* = 300) PICC for chemotherapy (*n* = 224)	Mean (range) 56 (1–487)	Completed therapy 22.9%, death 50.8%, unplanned removal 21.1%, patient request 0.5%, not assessed 1.9%	21.1	NR	Infection 0.18, phlebitis 3.84, broken catheter/leakage 1.97, accidental removal 1.18, occlusion 2.34 The complication rate was lower for phlebitis, leakage, and infection in palliative care. Higher for accidental removal	PICCs placed in cancer patients whether for chemotherapy or palliative care carry a low lethal complication rate	Weak
Yamada 2010, Japan [28]	Patient-Reported Usefulness of Peripherally Inserted Central Venous Catheters in Terminally Ill Cancer Patients	Case series (prospective) *n* = 39	Cancer patients admitted to a palliative care unit where a PICC was placed as part of regular care	PICC	Median (range) 15 (1–81)	Death 82%, occlusion 8%, accidental removal 10%, infection 0%	18	NR	NR	PICC use could be a safe, comfortable, and convenient method for terminally ill cancer patients, if the placement of a PICC was individualized to the patient situation and after the alternatives were considered	Moderate
Zhang 2011, China [29]	Persistent hiccup caused by peripherally inserted central catheter migration	Single case report	60-year-old male patient who was diagnosed with non-small cell lung cancer (NSCLC) with brain metastases	PICC	42	Catheter malposition with catheter folded in the jugular vein	NA	NR	NA	Persistent hiccup was in this case related to a PICC bent and folded in the right subclavian vein	Weak
Botella-Carretero 2013, Spain [16]	Role of Peripherally Inserted Central Catheters in Home Parenteral Nutrition: A 5-Year Prospective Study	Cohort study (prospective) *n* = 72	All patients attended at the Hospital Universitario Ramón y Cajal for home parenteral nutrition from 2007 to 2011	Hickman (*n* = 10) PICC (*n* = 48) Port (*n* = 21) PICC preferred for cancer patients or patients in need of HPN for < 6 months	Median (± IQR) 60 (± 129)	1 PICC removed due to a temperature and suspected infection	2.1	NR	PICC: Infection 0. No episodes of catheter-related thrombosis or thrombophlebitis. (No differences between Hickman and PICC)	PICCs may be considered an alternative to other central catheters even in patients with HPN for > 3 months and, in selected cases, > 6 months	Moderate
Bortolussi 2015, Italy [5]	Quality of Life, Pain Perception, and Distress Correlated to Ultrasound-Guided Peripherally Inserted Central Venous Catheters in Palliative Care Patients in a Home or Hospice Setting	Case series (prospective) *n* = 48	Adult patients with oncologic or degenerative disease referred to the Palliative Care Team in need of PICC or MC in a hospice or home setting. Life expectancy > 2 weeks	PICC (*n* = 13) MC (*n* = 35) (No information on selection criteria)	PICC median (range) 102 (13–462) MC median (range) 50.5 (8–231)	1 MC removed due to occlusion 1 PICC removed due to infection	7.7	NR (Only 7 of 48 patients alive at the end of the 16-month study period)	PICC and MC -total: occlusion 0.65, infection 0.32, accidental dislocation 0.65, leakage 0.65, inflammation 1.29	PICC or MC caused low level of pain and distress upon insertion, had low incidence of complications and favorable impact on quality of life	Moderate
Chopra 2015, USA [11]	The Michigan Appropriateness Guide for Intravenous Catheters (MAGIC): Results From a Multispecialty Panel Using the RAND/UCLA Appropriateness Method	Systematic review and guideline	Based on a systematic review including 219 articles, 665 scenarios for PICC indications were created. These scenarios were reviewed by 15 multispecialist panelists with the RAND/UCLA method, resulting in the current guideline	All CVC including PICC and MC	NA	NA	NA	NA	NA	Conclusion on the appropriate use of PICC in palliative care: PICC appropriate in palliative treatment during end-of-life care. Inappropriate for patient or family request in a patient who is not actively dying or in hospice, for comfort in obtaining daily blood samples for laboratory analysis	NA
Park K 2016, South Korea [27]	Safety, efficacy, and patient-perceived satisfaction of peripherally inserted central catheters in terminally ill cancer patients: a prospective multicenter observational study	Case series (prospective) *n* = 39 catheters 36 unique patients	Terminally ill cancer patients (expected survival less than 1–2 months)	PICC	Median (95%CI) 19 (14.1–23.9)	Death 72%, transfer or discharge 15%, accidental removal 12.8%	25.6	NR Overall median survival after admission to hospice: 39 days	Accidental removal 6.1, bleeding 3.7, thrombophlebitis 2.5	PICCs were safely inserted and showed a favorable maintenance rate with acceptable complications Additionally, most of the patients felt that parenteral access became more comfortable after PICC insertion. When considering the characteristics of terminally ill cancer patients, poor general condition, and a limited period of survival, PICC could be a safe and effective method for intravenous access	Weak
LoPriore 2017, Switzerland [20]	The role of a surveillance program for introducing peripherally inserted central catheters: a 2-year observational study in an academic hospital	Case series (prospective) *n* = 124	All patients who underwent PICC insertion from 1 January 2014, and had the catheter removed by 31 December 2015 at Bern University Hospital	PICC	(*N* = 124) Median (range) 62 (2–450) Patients with PICC for palliative care (*n* = 2) kept the catheter for 8 and 11 days	(*N* = 124 -both cancer (86.3%) and non-cancer patients.) End of treatment 63.7%, death 13.3%, PICC infection 5.2% (all cancer patients), PICC tip migration 5.2%, thrombosis 1.5%, occlusion 0.7%	12.6 (*n* = 124)	NR	(N = 124) Infection 0.48 (all cancer) Thrombosis 0.68 (6 of 7 cancer) Occlusion 0.68	Successful introduction of PICCs in an academic hospital by implementing a systematic surveillance program for complications. Both infectious and noninfectious complications were rare	Weak
Mori 2017, Japan [25]	Peripherally inserted central catheter-related bloodstream infection caused by *Kocuria marina* in an elderly man	Single case report	A 90-year-old Japanese man with sigmoid cancer and history of nasal cavity cancer and right cervical lymph node angiosarcoma	PICC	15	Catheter-related infection with *Kocuria marina*	NA	NR	NA	*K. marina* can be a causative pathogen in catheter-related bloodstream infections	Weak
Vashi 2017, USA [22]	Incidence of and factors associated with catheter-related bloodstream infection in patients with advanced solid tumors on home parenteral nutrition managed using a standardized catheter care protocol	Cohort study (retrospective) *n* = 408 catheters, 335 unique patients	Consecutive case series of cancer patients receiving HPN at Cancer Treatment Centers of America (CTCA) at Midwestern Regional Medical Center	PICC (*n* = 191) Port (*n* = 206) tCICC (*n* = 11) (selection based on hospital references)	Median 41	PICC infection 3.7% Other removal reasons not counted	3.7	NR	PICC-related infection 0.61	The incidence rate of CRBSI is low in an oncology population with HPN following a standardized catheter maintenance protocol. Type of VAD had no effect on the incidence of CRBSI	Moderate
Campagna 2019, Italy [17]	A retrospective study of the safety of over 100,000 peripherally inserted central catheters days for parenteral supportive treatments	Case series (retrospective) *n* = 1250	All adult inpatients and outpatients from four public hospitals who had a PICC inserted between September 2007 and December 2014 for the administration of parenteral supportive treatments and who had the PICC removed with removal date recorded 56.7% cancer patients	PICC	Median (IQR) 46 (19–120)	Death 54.1%, no further use 36.7%, accidental dislocation 9.1%, adverse events 14.2%	23.3	NR	Thrombosis 0.23, infection 0.32, occlusion 1.08, accidental dislocation 0.98	Low AE removal rate for PICC used to provide parenteral support for up to 6 months. Risk of PICC removal due to AE was higher with open‐system PICCs (HR 2.75)	Weak
Magnani 2019, Italy [6]	Peripherally inserted central catheter, midline, and "short" midline in palliative care: Patient-reported outcome measures to assess impact on quality of care	Case series (prospective) *n* = 90	Patients at palliative care unit (PCU) in need of VAD Type of VAD chosen according to international guidelines	PICC (*n* = 24) MC (*n* = 8) Short “MC” (9 ± 1 cm, inserted in superficial vein) (*n* = 58)	Patients were only followed for 14 days. (eight patients died within 3 days after catheter placement)	Accidental dislocation in 4 short MC Phlebitis in 5 short MC No episodes of occlusion or infection during 14-day follow-up	10	NR	NR	The insertion of a VAD in patients receiving palliative care resulted in a favorable impact on quality of care. The procedure was well tolerated and patients perceived the inserted VAD as useful and comfortable in most cases	Weak
Cotogni 2021, Italy [18]	Comparative Complication Rates of 854 Central Venous Access Devices for Home Parenteral Nutrition in Cancer Patients: A Prospective Study of Over 169,000 Catheter-Days	Cohort study (prospective) *n* = 854 central VADs 761 unique patients	All adult cancer outpatients who were candidates for HPN and had a central VAD inserted during the study period in a 1200-bed university hospital	PICC (*n* = 401) CICC (*n* = 137) tCICC (*n* = 118) port (*n* = 198) Selection based on clinicians’ preferences	PICC median (range) 194 (15–1154)	End of treatment 31%, death 64%, complications 5%	14.2	NR	PICC: local infection 0.07, CRBSI 0.08, DVT 0.08, mechanical complications 0.45	PICCs had significantly fewer CRBSIs and total complications than tCICC and were as safe and durable as ports	Moderate
Matysiak 2021, Poland [21]	Complications of tunneled peripherally inserted and tunneled-cuffed central catheters in home parenteral nutrition	Case series (prospective) n = 273 VADs 240 unique patients	Patients with type 3 chronic intestinal failure requiring HPN, 80% had cancer	tPICC (*n* = 150) tCICC (*n* = 123) tPICC preferred for an expected duration of HPN < 6 months	tPICC median (IQR) 119 (166) tCICC median (IQR) 324 (314)	14 of 150 (9%) tPICCs had to be removed due to complications CRBSI 3%, catheter damage 6%, accidental dislocation 0.7%	9	NR	tPICC: symptomatic thrombosis 0.17, asymptomatic thrombosis 0.09, mechanical damage 0.91, accidental dislocation 0.04, CRBSI 0.30	The tPICCs and tCICCs have a similar complication rate per 1000 catheter days, and tCICC can be safely replaced by tPICCs	Weak
Park EJ 2021, South Korea [26]	Safety, Efficacy, and Patient Satisfaction with Initial Peripherally Inserted Central Catheters Compared with Usual Intravenous Access in Terminally Ill Cancer Patients: A Randomized Phase II Study	Randomized controlled trial (unblinded) *n* = 57	Terminally ill cancer patients at a hospice palliative care (HPC) unit randomized to routine insertion of PICC at admission (*n* = 29) or usual IV access (*n* = 28)	PICC (*n* = 29) Usual IV access (*n* = 28)	Median (95% CI) 16 (8.5–23.5)	In routine PICC arm: death 79%, transfer 14%, accidental removal 7%	28.0	Median survival (Q1-Q3) from hospice admittance 16 (10.7–21.3) days	In routine PICC arm: catheter-related infection 1.6, thrombophlebitis 0, occlusion 0, bleeding 7.8, irritation 1.6, accidental removal 3.1	Routine PICC insertion in terminally ill cancer patients showed comparable safety and efficacy and superior satisfaction compared with usual IV access. Thus, routine PICC insertion could be considered at admission to the HPC unit	Moderate
Kopczynska 2022, UK [19]	Home Parenteral Nutrition in Patients with Advanced Cancer: Quality Outcomes from a Centralized Model of Care Delivery	Cohort study (retrospective) *n* = 126	Adults with advanced cancer requiring palliative HPN between 2010 and 2018 at a tertiary intestinal failure center	PICC (*n* = 36) Tunneled CICC (*n* = 90) Choice of catheter not specified	NR (Patient survival 80 days)	In total, 22.2% had one or more complications. (No info on catheter removal.) Occlusion 10%, catheter dislodgement 6.3%, CRBSI 3.2%, CVC thrombosis 0.8% No significant differences between catheter types		In total, 2.64 months (95% CI 2.17–3.38)	CRBSI 0.49, all in the CICC group	8.5% of patients were readmitted due to HPN complications; 34.9% of patients died at home. No correlation between CRBSI or mechanical catheter complications and age, sex, CVC type, no. lumen, chemotherapy, venting tube present, or distance from home to hospital	Moderate

*AE* adverse Events, *CICC* centrally inserted central catheter, *CRBSI* catheter-related bloodstream infection, *CVC* central vascular catheter, *DVT* deep vein thrombosis, *EPHPP global rating* Effective Public Health Practice Quality Global rating score, *HPN* home parenteral nutrition, *IV* intravenous, *MC* midline catheter, *NA* not applicable, *NR* not reported, *PICC* peripherally inserted central catheter, *RAND/UCLA* Research And Development Corporation/University of California, Los Angeles, *tCICC* tunneled centrally inserted central catheter, *tPICC* tunneled peripherally inserted central catheter, *VAD* vascular access device

Six studies were carried out on patients in palliative care units or hospices [5, 6, 24, 26–28], of which two studies also encompassed home care settings [5, 24]. Five studies were conducted on cancer patients receiving home parenteral nutrition (HPN) [16, 18, 19, 21, 22]. Three studies were undertaken on patients from several hospital wards, or hospitals with mixed patient populations where 56–100% had cancer [17, 20, 23]. The two single case studies described hospitalized palliative care cancer patients with catheter-related complications [25, 29]. The number of participants per study ranged from 1 to 1250. In two studies, the use of both PICC and MC was investigated [5, 6]; in nine studies, exclusively PICC was addressed [17, 20, 23–29]; and in six studies, PICC was examined in comparison to other central venous catheters [11, 16, 18, 19, 21, 22].

### Quality assessment

The EPHPP global rating scores are presented in Table 1. None of the included articles were rated “strong.” Seven were rated “moderate” and nine as “weak.” The most common factors contributing to a weak rating were the lack of blinding, failure to control for confounders, and the use of unvalidated data collection methods.

### Indications for catheter placement and catheter utilization

The six studies conducted primarily in palliative care units and hospices [5, 6, 24, 26–28] all documented pain or symptom management as an indication for PICC or MC. Additional indications reported in these studies included hydration, blood sampling, blood product transfusions, and nutrition.

Five studies were primarily concerned with PICC utilized for HPN in cancer patients [16, 18, 19, 21, 22]. In two of these studies [16, 21], PICCs or tunneled PICCs were by local tradition preferred to centrally inserted catheters and ports in patients with low-performance status, palliative treatment intention, and expected period of HPN shorter than 6 months. In the other three studies on patients with HPN, either no selection criteria were presented, or clinicians’ and institutional preferences were described as motivating factors for the choice of PICC or other central venous access options. In the three studies from mixed hospital wards [17, 20, 23], catheter indications such as antibiotics, blood sampling and blood products, nutrition, hydration, pain management, and palliative care were reported.

### PICC and MC dwell times and patient survival after catheter placement

Median dwell times for PICCs were documented in 11 studies and ranged from 15 to 194 days across studies. PICC dwell time of less than 20 days was reported in four studies [23, 26–28]. Three of these studies were conducted in palliative care units where death was the reason for catheter removal in 72–82% of cases. In a study mainly on patients receiving chemotherapy, but with a small subgroup of two patients who received a PICC primarily for palliative care [20], the PICC dwell times for these two patients were 8 and 11 days, respectively.

Three studies demonstrated a median PICC dwell time of more than 100 days [5, 18, 21]. Two of these studies were conducted on cancer patients with PICCs utilized for HPN. The third study was from a palliative care network of hospices and home care services and included 13 patients with PICCs that had a median dwell time of 102 (range 13–462) days [5]. Removal reasons were not specified, but at the end of the 17-month study period, 11 of 13 patients with PICC had died. This study was also the only one that presented an MC median dwell time, which for the 42 participants was 50.5 (range 8–231) days. Five of the 42 patients were still alive at the end of the study.

The other study including MCs was performed within a palliative care department [6] and encompassed eight patients with MCs and 58 patients with short (9 cm) MCs. At the end of the study, 44% of the participants had dropped out. The proportion of patients who died during the 14-day study period is not disclosed, but already at follow-up day three, 9% of participants were deceased.

Patient survival after catheter placement was reported explicitly in only one study [19]. In this British study on HPN for patients with advanced cancer, the median overall survival after PICC or centrally inserted tunneled catheters was 80 days (2.64 months). In addition, two studies from South Korea reported median survival of 16 and 39 days after hospital admittance for PICC [26, 27]. Both studies were conducted on terminally ill cancer patients admitted to hospice or palliative care departments.

### Reasons for removal of PICC and MC

Four studies on patients in hospices and palliative care units [5, 26–28] and one study on cancer patients receiving HPN [18] described death as the main reason for catheter removal. The latter study also had the longest median PICC dwell time of the included studies. Additionally, the three studies on mixed patient populations [17, 20, 23] reported death as the reason for PICC removal in 13.3–54.1% of cases. In the same three studies, completed therapy was the removal reason in 36.7–63.7% of cases. The Italian study on cancer patients receiving HPN [18] and the Taiwanese study comparing PICC for palliative care with PICC for chemotherapy [24] reported completed therapy as reason for PICC removal in 31% and 22.9% of cases, respectively.

All of the clinical studies on PICC described complications as a reason for catheter removal, accounting for 1–33% of cases. Accidental removal was documented in eight studies [17, 19, 21, 23, 24, 26–28] with a frequency of 0.7–12.3%. Occlusion as removal reason was outlined in four studies [5, 20, 24, 28] with 0.7–8% frequency. Infection-related PICC removal was reported in nine studies with a frequency range of 0–8% [5, 6, 16, 19–22, 25, 28]. Catheter removal due to catheter-related thrombosis was noted in two studies, with 1.5 and 4% frequencies [20, 23]. One study reported catheter removal in 5.2% of patients due to catheter tip migration [20], and another study reported catheter damage in 6% of PICCs [21]. One study mentioned patients’ requests as the reason for 0.5% of PICC removals [24]. In the RCT from South Korea conducted in a palliative care unit, PICC removal due to patient transfer to home or other hospitals was reported in 14% of PICC users [26].

MC removal due to complications was reported in two Italian studies [5, 6]. In the study on palliative care patients in hospice or home care [5], one of the 35 study participants had the MC removed due to occlusion. In the 14-day follow-up study including eight patients with MC and 58 patients with short MC in a palliative care unit [6], four patients had an accidental removal of a short MC, and five patients with a short MC placed in a superficial vein had phlebitis.

The two single case studies described complications to PICC in palliative care patients with cancer [25, 29]. One was a case of persistent hiccups, in which the PICC had migrated to and was bent and folded in the right subclavian vein. The symptom resolved after catheter removal. The other case was a patient with intestinal obstruction after abdominal cancer surgery and PICC-related infection with *Kocuria marina*, which was resolved after catheter removal and antimicrobial therapy.

### PICC and MC complication rates

The overall complication rate for PICC was documented in eight studies and ranged from 0.69 to 14.1 per 1000 catheter days [5, 17, 18, 20, 21, 23, 24, 27]. There were no reports on complication rates for MC, but in one study on 13 PICCs and 35 MCs, the overall complication rate was 3.6 per 1000 catheter days.

In some of the studies, only complications leading to catheter removal were counted [17, 23, 24]. In one study, only the complication of catheter-related bloodstream infections was investigated [22]. Two studies omitted any mentioning of (accidental) catheter dislocation as a complication [16, 20]. It is possible that either no instances of accidental catheter removal occurred, or this issue was not regarded as a complication in those studies.

Catheter-related bloodstream infections were reported in eleven studies [5, 16–24, 26], with a rate between 0 and 2.46 per 1000 catheter days. A catheter-related infection was commonly defined as a clinically suspected bloodstream infection verified by positive peripheral and central blood culture or catheter tip culture [5, 16, 18, 21, 22, 26]. Two studies registered suspected catheter-related infections unverified by blood or tip culture [20, 23], one study only registered suspected exit site infections [17], and two studies did not provide a definition [19, 24].

The highest complication rate was from the 2002 US study, which included 351 PICCs in cancer patients of all ages [23]. This study included both primary and secondary catheter-related bloodstream infections and the highest rates were found among patients with hematological malignancies and bone marrow transplants. Eight studies reported a rate below 0.5 per 1000 catheter days, including four studies primarily focusing on HPN [16, 18, 19, 21], two studies on palliative care cancer patients with PICC for symptom treatment [5, 24], and two studies with mixed populations and indications for PICC [17, 20]. Neither of the two studies on MC identified MC-related bloodstream infections [5, 6].

The rate of PICC-related thrombosis ranged from 0 to 1.14 per 1000 catheter days [16–18, 20, 21, 23, 26]. Typically, a radiology-verified diagnosis of thrombosis required the participant to exhibit symptoms. However, in the Polish study on HPN, routine ultrasound investigations detected both symptomatic and asymptomatic deep vein thrombosis at a rate of 0.17 and 0.09 per 1000 catheter days, respectively [21]. MC-related thrombosis was not recognized in any of the studies.

PICC occlusion varied from 0 to 2.34 per 1000 catheter days [5, 17, 20, 23, 24, 27]. Occlusion was commonly acknowledged as a lumen occlusion resistant to attempts of regaining flow. In one of the larger retrospective case reviews with an occlusion rate of 1.08 per 1000 catheter days, both complete and partial occlusion and withdrawal occlusion were considered [17].

Accidental removal occurred at a rate of 0.04 to 6.1 per 1000 PICC days [5, 17, 21, 23, 24, 26, 27]. The lowest rate was reported in the Polish study where PICCs were tunneled [21]. The highest rates of 3.1 and 6.1 were documented in the two South Korean studies from palliative care units where median patient survival was 16 and 39 days, respectively [26, 27]. In both studies, an association with terminal delirium was suggested.

### Palliative care *cancer* patients’ experiences with PICC or MC

Patient-related outcome measures were only documented in studies from hospice/palliative care departments [5, 26–28]. In these studies, 79–94% of patients reported little or no distress during PICC procedure [27, 28], and 83–96% of patients reported a favorable satisfaction with PICC compared to before PICC [27, 28] or compared to peripheral vascular access [26]. In three of these studies [26–28], assessments of patient satisfaction were conducted in an unblinded manner with study-specific questionnaires. Participants were asked after catheter placement, “Did you experience distress because of the procedure?” They could respond with “distressing,” “slightly distressing,” or “not distressing.” In two of these studies [26, 27], participants were also asked 5 days after catheter placement, “Are you more comfortable after placement of parenteral access?” Participants could answer on a verbal rating scale ranging from “much comfort,” to “much discomfort.”

In a study on PICC and MC for palliative care patients in hospice or home care [5], quality of life was assessed with the European Organization for Research and Treatment of Cancer Quality of Life Questionnaire Core15-Palliative (EORTC QLQ-C15-PAL) [30], which was completed at baseline and 1 week after catheter placement. The study demonstrated improved global health score 1 week after the procedure, along with a trend of improved scores on physical symptoms. In another study on PICC or MC impact on quality of care for patients at a palliative care unit, the Palliative Care Outcome Scale (POS) indicated a favorable impact on quality of care 3-day post-catheter placement [6]. It is worth noting that these improvements might also be attributed to the simultaneous initiation of specialist palliative care. There were no differences between PICC and MC subgroups.

## Discussion

This systematic review included 17 articles concerning the application of PICC and MC for supportive interventions and symptom treatment in palliative care cancer patients, with the majority of studies being case series and cohort studies. Strikingly, only three of the included studies actually incorporated MCs. A mere six of the included studies were based in specialist palliative care settings, emphasizing the limited evidence surrounding both the use and the complications of PICCs in this setting.

The PICC dwell times exhibited considerable variation across studies, which also reflects when in the patient’s disease trajectory the catheter was placed: The duration of the PICC usage was protracted when used for HPN, while the typical duration for pain and symptom management was 2–4 weeks, with the catheter often maintained until death. This pattern may underscore the heightened need for parenteral symptom treatment as patients near the end of life. Moreover, the broad variation in dwell times of PICCs, including shorter durations than an MC typically could accommodate, suggests the absence of clear guidelines on choosing the most fitting vascular access for these scenarios. Notably, even in the study where the median dwell time for MCs was 50.2 days—surpassing the recommended 30-day lifespan—the complication rates remained low [5].

Our review found PICCs and MCs to have a generally low complication rate. Notably, one of the highest rates was from the oldest study, which saw more frequent infections and phlebitis [23]. In this study, diverse catheter placement methods, often without ultrasound guidance, were practiced. One important reason for variation in complication rates across studies was discrepancies in what was considered a complication. Some studies focused solely on complications leading to catheter removal, and complications such as accidental catheter dislocation and bleeding were inconsistently recorded. Notably, trivial bleeding during catheter insertion was reported only in the two South Korean studies [26, 27], contributing to their elevated complication rates.

The rates for catheter-related thrombosis, infections, and occlusion were all low (0–2.46 per 1000 catheter days), underlining the safety of these catheters. In terminally ill patients, diagnostic procedures might be skipped to spare discomfort, leading to underreporting of complications. However, in several of the included studies, there were attempts to compensate this through routine PICC culture after catheter removal [26–28] or routine ultrasound to detect asymptomatic catheter-related thrombosis [21].

Short catheter dwell times might influence observed complications, as risk factors for complications, such as infections, amplify with extended use of central venous catheters. However, in the included studies, no discernible trend of rising infection rates was evident with extended dwell times.

Cancer patients with PICCs utilized primarily for chemotherapy were not encompassed in this review. These patients face, especially when immunocompromised, an elevated risk of catheter-related infections [31]. This assertion is substantiated by one of the studies within our review which demonstrated a higher infection rate in PICCs used for chemotherapy compared to PICCs used for symptom management [24].

Previous studies have indicated an association between catheter-related thrombosis and cancer, particularly hematological cancers, when using PICCs [32]. However, advancements in insertion techniques in recent research appear to mitigate this risk [33]. Furthermore, it is suggested that catheter diameter, especially multi-lumen catheters, may elevate thrombosis risk [34]. The majority of studies in our review exclusively utilized single-lumen PICCs. In the reviewed studies, ports or other central venous catheters (CVCs) were typically favored over PICCs for chemotherapy and anticipated longer catheter dwell time, which might influence the observed complication rates.

Patient satisfaction regarding PICC or MC was uniformly high. This is in contrast to findings from a study involving a broader cohort of hospitalized patients, who, when receiving PICC or MC for various reasons, reported higher levels of distress from both the procedure and the indwelling catheter [35]. The consistent positive response in our reviewed studies might be influenced by the non-blinded nature of evaluations and potentially further biased by feedback being provided directly to the treating clinician. In the included studies, patient satisfaction with the catheter was assessed in comparison to peripheral venipuncture, and the timing of evaluation often coincided with admittance to specialist palliative care. Furthermore, introducing a comparison to optimized subcutaneous symptom management could alter perceptions.

Despite the detailed review, there were inherent limitations. Relevant papers may have been published after the literature search was completed. Defining the palliative care cancer population is a challenge. To counteract this, we employed a previously published search strategy specifically curated to have a high sensitivity for identifying palliative care patients and cast a wide net by including both “cancer” and “palliative care” patients in our search strategy. Palliative care is applicable at different stages of the cancer disease trajectory, which may result in large variations in catheter utilization, dwell times, and complications within this heterogeneous population. Parenteral nutrition, if indicated, is typically provided earlier in the disease trajectory and may present different catheter complications from those seen with pain and symptom management. Including studies with parenteral nutrition could therefore be questioned. However, the complication rates remained low for this intervention in palliative care patients. The few relevant hits, low quality of evidence, and the broad spectrum of indications and settings highlight a need for additional research to elucidate the role of PICC and MC in the management of palliative care cancer patients.

## Conclusion

PICC and MC are safe tools with high user satisfaction and can be utilized for extended periods. However, we still lack clarity on optimal deployment for pain and symptom management in palliative care cancer patients, specifically in relation to preferential use over peripheral IV or SC administration routes. Questions also remain regarding the optimal timing in a patient’s disease trajectory to introduce these catheters and when to favor PICC over MC, especially if a catheter is only required for a few weeks. Further studies are needed to clarify indications for PICC or MC in palliative care.

### Supplementary Information

Below is the link to the electronic supplementary material.

Supplementary file1 (DOCX 23 KB)

## Data Availability

No datasets were generated or analyzed during the current study.
