# Supplementary material for: Use of peripherally inserted central venous catheters and midline catheters for palliative care in patients with cancer: a systematic review
Source: Support Care Cancer. 2024 Jun 26;32(7):464. doi: 10.1007/s00520-024-08664-3 (PMC11208250; doi:10.1007/s00520-024-08664-3)
Supplement: Supplementary file 1 — Supplementary file1 (DOCX 23 KB) [file 520_2024_8664_MOESM1_ESM.docx]

| **Title** | Use of peripherally central venous catheters and midline catheters in palliative care cancer patients. A systematic review |
| --- | --- |
| **PICO** | P: palliative care cancer patients  I: PICC or MC (for symptom management in palliative care)  C:  O: |
| **Contact information** | Name: Eva Gravdahl  E-mail: Eva.Gravdahl@ahus.no  Phone: 40204004 |
| **Librarian** | Navn: Helene Lie  Akershus University Hospital  E-mail: heli@ahus.no  Phone: 67968562 |

**Systematic Reviews**

| **Database** | [**The Cochrane Library**](http://onlinelibrary.wiley.com/cochranelibrary/search/) |
| --- | --- |
| **Date searched** | 23.12.2022 |
| **Search history** | Date Run: 23/12/2022 05:17:29  #1 MeSH descriptor: [Neoplasms] explode all trees 90536  #2 MeSH descriptor: [Carcinoma] explode all trees 15236  #3 MeSH descriptor: [Medical Oncology] explode all trees 270  #4 ((neoplas* or cancer* or tumor* or tumour* or onco* or carcinom* or malignan* or metastas*)):ti,ab,kw (Word variations have been searched) 258040  #5 #1 or #2 or #3 or #4 267684  #6 MeSH descriptor: [Terminal Care] explode all trees 523  #7 MeSH descriptor: [Terminally Ill] this term only 94  #8 MeSH descriptor: [Palliative Care] explode all trees 1805  #9 MeSH descriptor: [Palliative Medicine] explode all trees 2  #10 MeSH descriptor: [Hospice Care] explode all trees 118  #11 MeSH descriptor: [Hospice and Palliative Care Nursing] explode all trees 47  #12 ((terminal* or end-stage or endstage or advanced) NEAR/3 (care or disease* or ill*)):ti,ab,kw (Word variations have been searched) 15405  #13 ((palliat* or hospice*)):ti,ab,kw (Word variations have been searched) 9732  #14 (close NEAR/3 death):ti,ab,kw (Word variations have been searched) 88  #15 (("end of life" or EOL or terminal-stage* or terminalstage* or terminal-phase* or dying or "cancer pain")):ti,ab,kw (Word variations have been searched) 23864  #16 (advance NEAR/2 (care or health care or healthcare)):ti,ab,kw (Word variations have been searched) 1645  #17 (morphine AND (cancer or neoplasm*)):ti,ab,kw (Word variations have been searched) 1818  #18 #6 OR #7 OR #8 OR #9 OR #10 OR #11 OR #12 OR #13 OR #14 OR #15 OR #16 OR #17 46089  #19 #5 OR #18 292910  #20 MeSH descriptor: [Vascular Access Devices] explode all trees 434  #21 MeSH descriptor: [Central Venous Catheters] explode all trees 202  #22 MeSH descriptor: [Catheterization, Central Venous] explode all trees 900  #23 MeSH descriptor: [Catheters, Indwelling] explode all trees 1059  #24 MeSH descriptor: [Catheterization, Peripheral] explode all trees 1090  #25 ((PICC or PICCs)):ti,ab,kw (Word variations have been searched) 446  #26 (peripheral* NEAR/4 (catheter* or cannula* or insert* or implant* or access or device*)):ti,ab,kw (Word variations have been searched) 2822  #27 ("extended dwell"):ti,ab,kw (Word variations have been searched) 5  #28 extended dwell 30  #29 ((midline* or mid-line* or medium-long or medium-term) NEAR/5 (catheter* or cannula* or insert* or implant* or access or device*)):ti,ab,kw (Word variations have been searched) 217  #30 #20 OR #21 OR #22 OR #23 OR #24 OR #25 OR #26 OR #27 OR #28 OR #29 4947  #31 #19 AND #30 709  #31         #19 AND #30       709  #32        #19 AND #30  *in Cochrane Reviews* 22  #33        #19 AND #30  *in CENTRAL* 687 |
| **Hits** | Cochrane Reviews (22)  Cochrane Central Register of Controlled Trials (CENTRAL) (687) |

**Primary Studies**

| **Database** | MEDLINE  Oppgi valgt tidsspenn i alle databaser |
| --- | --- |
| **Date searched** | 09.12.2022 |
| **Search history** | Search for: limit 21 to (danish or english or norwegian or swedish)  Results: 10  Database: Ovid MEDLINE(R) ALL <1946 to December 06, 2022> Search Strategy:  --------------------------------------------------------------------------------  1 exp Neoplasms/ or exp Carcinoma/ or Medical Oncology/ (3773261)  2 (neoplas* or cancer* or tumor* or tumour* or onco* or carcinom* or malignan* or metastas*).ab,kf,ti. (3995819)  3 1 or 2 (4995179)  4 exp Terminal Care/ or Terminally Ill/ or Palliative Care/ or Palliative Medicine/ or Hospice Care/ or "Hospice and  Palliative Care Nursing"/ (108617)  5 ((terminal* or end-stage or endstage or advanced) adj3 (care or disease* or ill*)).ab,kf,ti. (127092)  6 (palliat* or hospice*).ab,kf,ti. (99986)  7 (close adj3 death).ab,kf,ti. (747)  8 ("end of life" or EOL or terminal-stage* or terminalstage* or terminal-phase* or dying or cancer pain).ab,kf,ti.  (78455)  9 (advance adj2 (care or health care or healthcare)).ab,kf,ti. (5810)  10 (morphine and (cancer or neoplasm*)).ab,kf,ti. (3800)  11 4 or 5 or 6 or 7 or 8 or 9 or 10 (320120)  12 3 or 11 (5188925)  13 Vascular Access Devices/ or Central Venous Catheters/ or Catheterization, Central Venous/ or Catheters,  Indwelling/ or Catheterization, Peripheral/ (43265)  14 (PICC or PICCs).mp. (1806)  15 (peripheral* adj4 (catheter* or cannula* or insert* or implant* or access or device*)).ab,kf,ti. (8732)  16 extended dwell.mp. (26)  17 ((midline* or mid-line* or medium-long or medium-term or medial) adj5 (catheter* or cannula* or insert* or  implant* or access or device*)).ab,kf,ti. (3365)  18 13 or 14 or 15 or 16 or 17 (51522)  19 12 and 18 (7618)  20 limit 19 to yr="2000 -Current" (5346)  21 20 not ((exp infant/ or exp child/ or adolescent/) not exp adult/) (4684)  22 limit 21 to (danish or english or norwegian or swedish) (4222) |
| **Hits** | 4222 |
| **Comments** |  |

| **Database** | EMBASE |
| --- | --- |
| **Date searched** | 23.12.2022 |
| **Search history** | Search for: 22 not ((exp infant/ or exp child/ or adolescent/) not exp adult/)  Results: 629  Database: Embase <1974 to 2022 December 22>  Search Strategy:  --------------------------------------------------------------------------------  1 exp neoplasm/ (5273042)  2 exp carcinoma/ (1346445)  3 exp oncology/ (178296)  4 (neoplas* or cancer* or tumor* or tumour* or onco* or carcinom* or malignan* or metastas*).ab,kf,ti. (5301913)  5 1 or 2 or 3 or 4 (6554451)  6 exp terminal care/ or terminally ill patient/ or exp palliative therapy/ or palliative nursing/ (197355)  7 ((terminal* or end-stage or endstage or advanced) adj3 (care or disease* or ill*)).ab,kf,ti. (198947)  8 (palliat* or hospice*).ab,kf,ti. (160349)  9 (close adj3 death).ab,kf,ti. (1024)  10 ("end of life" or EOL or terminal-stage* or terminalstage* or terminal-phase* or dying or cancer pain).ab,kf,ti.  (110844)  11 (advance adj2 (care or health care or healthcare)).ab,kf,ti. (9404)  12 (morphine and (cancer or neoplas*)).ab,kf,ti. (6495)  13 6 or 7 or 8 or 9 or 10 or 11 or 12 (497458)  14 5 or 13 (6833511)  15 exp peripherally inserted central venous catheter/ (4570)  16 (PICC or PICCs).mp. (4037)  17 (peripheral* adj4 (catheter* or cannula* or insert* or implant* or access or device*)).ab,kf,ti. (14031)  18 extended dwell.mp. (51)  19 ((midline* or mid-line* or medium-long or medium-term) adj5 (catheter* or cannula* or insert* or implant* or  access or device*)).ab,kf,ti. (1580)  20 15 or 16 or 17 or 18 or 19 (18087)  21 14 and 20 (3286)  22 limit 21 to (english language and yr="2000 -Current") (2935)  23 22 not ((exp infant/ or exp child/ or adolescent/) not exp adult/) (2628) |
| **Hits** | 2628 |
| **Comments** |  |

| **Database** | Web of Science |
| --- | --- |
| **Date searched** | 19.12.2022 |
| **Search history** | # Web of Science Search Strategy (v0.1)  # Database: Web of Science Core Collection  # Entitlements:  - WOS.SCI: 1945 to 2022  - WOS.AHCI: 1975 to 2022  - WOS.ESCI: 2017 to 2022  - WOS.SSCI: 1956 to 2022  # Searches:  **#1  (TS=((neoplas* or cancer* or tumor* or tumour* or onco* or carcinom* or malignan* or metastas*)))**  **#2  ALL=((morphine and (cancer or neoplasm*)))**  **#3  TS=(advance NEAR/2 care or health care or healthcare)**  **#4  TS=(("end of life" or EOL or terminal-stage* or terminalstage* or terminal-phase* or dying or "cancer pain"))**  **#5  TS=(close NEAR/3 death)**  **#6  TS=((palliat* or hospice*))**  **#7  TS=(((terminal* or end-stage or endstage or advanced) NEAR/3 (care or disease* or ill*)))**  **#8  #2 OR #3 OR #4 OR #5 OR #6 OR #7**  **#9  #1 OR #8**  **#10 TS=((midline* or mid-line* or medium-long or medium-term) NEAR/5 (catheter* or cannula* or insert* or implant* or access or device*))**  **#11  TS=("extended dwell")**  **#12  TS=(PICC or PICCs)**  **#13  TS=( (peripheral* NEAR/4 (catheter* or cannula* or insert* or implant* or access or device*)))**  **#14  #10 OR #11 OR #12 OR #13**  **#15  #9 AND #14**  **#16 #15** Timespan: 2000-01-01 to 2022-12-31 and **English** (Languages) |
| **Hits** | 1791 |
| **Comments** |  |

| **Database** | CINAHL |
| --- | --- |
| **Date searched** | 22.12.2022 |
| **Search history** | \|  \| Thursday, December 22, 2022 3:59:45 PM \| \| --- \| --- \|  \| **#** \| **Query** \| **Limiters/Expanders** \| **Results** \| \| --- \| --- \| --- \| --- \| \| S31 \| S17 AND S27 \| Limiters - Published Date: 20000101-20231231 Narrow by SubjectAge: - all adult Narrow by Language: - english Search modes - Boolean/Phrase \| 1,291 \| \| S30 \| S17 AND S27 \| Limiters - Published Date: 20000101-20231231 Narrow by Language: - english Search modes - Boolean/Phrase \| 3,284 \| \| S29 \| S17 AND S27 \| Limiters - Published Date: 20000101-20231231 Search modes - Boolean/Phrase \| 3,488 \| \| S28 \| S17 AND S27 \| Search modes - Boolean/Phrase \| 3,872 \| \| S27 \| S18 OR S19 OR S20 OR S21 OR S22 OR S23 OR S24 OR S25 OR S26 \| Search modes - Boolean/Phrase \| 26,036 \| \| S26 \| TX (midline* or mid-line* or medium-long or medium-term) N5 (catheter* or cannula* or insert* or implant* or access or device*) \| Search modes - Boolean/Phrase \| 453 \| \| S25 \| TX extended dwell \| Search modes - Boolean/Phrase \| 28 \| \| S24 \| TX (peripheral* N4 (catheter* or cannula* or insert* or implant* or access or device*) \| Search modes - Boolean/Phrase \| 7,998 \| \| S23 \| TX (PICC or PICCs) \| Search modes - Boolean/Phrase \| 1,629 \| \| S22 \| (MH "Catheterization, Peripheral") \| Search modes - Boolean/Phrase \| 3,948 \| \| S21 \| (MH "Catheters+") \| Search modes - Boolean/Phrase \| 15,395 \| \| S20 \| (MH "Catheterization, Central Venous+") \| Search modes - Boolean/Phrase \| 5,632 \| \| S19 \| (MH "Central Venous Catheters+") \| Search modes - Boolean/Phrase \| 4,593 \| \| S18 \| (MH "Vascular Access Devices+") \| Search modes - Boolean/Phrase \| 9,256 \| \| S17 \| S5 OR S16 \| Search modes - Boolean/Phrase \| 1,239,688 \| \| S16 \| S6 OR S7 OR S8 OR S9 OR S10 OR S11 OR S12 OR S13 OR S14 OR S15 \| Search modes - Boolean/Phrase \| 183,921 \| \| S15 \| TX morphine and (cancer or neoplasm*) \| Search modes - Boolean/Phrase \| 2,460 \| \| S14 \| TX advance N2 (care or "health care" or healthcare) \| Search modes - Boolean/Phrase \| 12,075 \| \| S13 \| TX ("end of life" or EOL or terminal-stage* or terminalstage* or terminal-phase* or dying or "cancer pain") \| Search modes - Boolean/Phrase \| 52,261 \| \| S12 \| TX close N3 death \| Search modes - Boolean/Phrase \| 461 \| \| S11 \| TX (palliat* or hospice*) \| Search modes - Boolean/Phrase \| 102,896 \| \| S10 \| TX (terminal* or end-stage or endstage or advanced) N3 (care or disease* or ill*) \| Search modes - Boolean/Phrase \| 67,764 \| \| S9 \| (MH "Hospice Care") \| Search modes - Boolean/Phrase \| 9,749 \| \| S8 \| (MH "Palliative Care") OR (MH "Hospice and Palliative Nursing") OR (MH "Palliative Medicine") \| Search modes - Boolean/Phrase \| 41,567 \| \| S7 \| (MH "Terminally Ill Patients+") \| Search modes - Boolean/Phrase \| 12,780 \| \| S6 \| (MH "Terminal Care+") \| Search modes - Boolean/Phrase \| 72,524 \| \| S5 \| S1 OR S2 OR S3 OR S4 \| Search modes - Boolean/Phrase \| 1,120,321 \| \| S4 \| TX (neoplas* or cancer* or tumor* or tumour* or onco* or carcinom* or malignan* or metastas*) \| Search modes - Boolean/Phrase \| 1,080,426 \| \| S3 \| (MH "Oncology+") \| Search modes - Boolean/Phrase \| 12,756 \| \| S2 \| (MH "Carcinoma+") \| Search modes - Boolean/Phrase \| 102,700 \| \| S1 \| (MH "Neoplasms+") \| Search modes - Boolean/Phrase \|  \| |
| **Hits** | 1291 |
| **Comments** |  |
